# A Systematic Review for Anti-Inflammatory Property of Clusiaceae Family: A Preclinical Approach

**DOI:** 10.1155/2014/960258

**Published:** 2014-05-26

**Authors:** Mônica Santos de Melo, Jullyana de Souza Siqueira Quintans, Adriano Antunes de Souza Araújo, Marcelo Cavalcante Duarte, Leonardo Rigoldi Bonjardim, Paulo Cesar de Lima Nogueira, Valéria Regina de Souza Moraes, João Xavier de Araújo-Júnior, Êurica Adélia Nogueira Ribeiro, Lucindo José Quintans-Júnior

**Affiliations:** ^1^Department of Physiology, Federal University of Sergipe, São Cristóvão, SE, Brazil; ^2^Department of Pharmacy, Federal University of Sergipe, São Cristóvão, SE, Brazil; ^3^Bauru School of Dentistry, University of São Paulo, Bauru, SP, Brazil; ^4^Department of Chemistry, Federal University of Sergipe, São Cristóvão, SE, Brazil; ^5^School of Nursing and Pharmacy, Federal University of Alagoas, Maceió, AL, Brazil; ^6^Laboratory of Pre-Clinical Pharmacology (LAPEC), Department of Physiology, Federal University of Sergipe (UFS), São Cristóvão, SE, Brazil

## Abstract

*Background.* Clusiaceae family (sensu lato) is extensively used in ethnomedicine for treating a number of disease conditions which include cancer, inflammation, and infection. The aim of this review is to report the pharmacological potential of plants of Clusiaceae family with the anti-inflammatory activity in animal experiments. *Methods.* A systematic review about experiments investigating anti-inflammatory activity of Clusiaceae family was carried out by searching bibliographic databases such as Medline, Scopus and Embase. In this update, the search terms were “anti-inflammatory agents,” “Clusiaceae,” and “animals, laboratory.” *Results.* A total of 255 publications with plants this family were identified. From the initial 255 studies, a total of 21 studies were selected for the final analysis. Studies with genera *Allanblackia*, *Clusia*, *Garcinia* or *Rheedia*, and *Hypericum* showed significant anti-inflammatory activity. The findings include a decrease of total leukocytes, a number of neutrophils, total protein concentration, granuloma formation, and paw or ear edema formation. Other interesting findings included decreased of the MPO activity, and inflammatory mediators such as NF-**κ**B and iNOS expression, PGE_2_ and Il-1**β** levels and a decrease in chronic inflammation. *Conclusion.* The data reported suggests the anti-inflammatory effect potential of Clusiaceae family in animal experiments.

## 1. Introduction

Inflammation is a process that occurs after an infection or tissue injury, characterized by increased postcapillary venule permeability to fluid and plasma proteins and polymorphonuclear leukocyte emigration into tissues [1]. The inflammatory response is essential in maintaining homeostasis; however, this event may be chronic course, leading to tissue damage due to leukocytosis, fibroplasia, excessive production of cytokines, and other mediators [2]. Generally, anti-inflammatory drugs, such as nonsteroidal anti-inflammatory drugs, are effective for temporary relief of symptoms. However, drug-induced severe side effects occur, and most of these treatments are inadequate for chronic use [3].

Many people turn to alternative medicine including traditional plant based remedies for alleviating inflammatory conditions, such as plant-derived extracts or plant derivatives (isolated compounds), by controlling the levels of various inflammatory cytokines or inflammatory mediators [4–6]. The effect of medicinal plants is mediated by multiple targets through multiple active compounds [6, 7]. Although source around the world has made studies on the anti-inflammatory studies from different pathways and aspects and has made substantial progress, further studies are warranted to delineate the inflammation actions in more cogency models, assess the potentials in clinical applications, and make more convenient preparations easy to administrate for patients [8].

In this context, several ethnobotanical studies have reported the bioprospecting surveys on the positive use of Clusiaceae family with pharmacological activity [9]. Consequently, these plant species have received attention from the scientific community for its potential therapeutic capacity. Clusiaceae is a tropical family of trees, shrubs, and herbs comprising approximately 50 genera and 1200 species [10]. Several species of this family are used for medicinal purposes worldwide, as for the treatment of cancer, inflammation, and infection. In Brazil, the most described genera are* Kielmeyera* Mart. & Zucc.,* Caraipa* Aubl.,* Platonia* Mart.,* Clusia* L.,* Rheedia* L., and* Calophyllum* L. [3–6].

The Clusiaceae family is a rich source of secondary metabolites, in which four major classes of compounds are found: xanthones, coumarins, biflavonoids, and benzophenones, produced by the plants mainly as a defense mechanism [9]. Despite the importance of this family experimental research on the anti-inflammatory effect with preparations on plants of Clusiaceae family has never been reviewed.

Although a lot of important information or clues on the development of inflammation can be obtained from human studies, animal models not only enable us to have a more comprehensive understanding of the inflammation at a molecular level in a controlled manner, but also fulfill the need for drug screening tools. This not only allows a faster and more convenient screening but also serves as an alarm before the presence of cellular or functional lesion. Based on the mechanistic studies, drugs targeting different molecules in the cascade are being developed. In order to evaluate the effect of the drug properly, reliable and appropriate animal models are required. Therefore, in this review, we focus on the animal models of inflammation that researchers have used. So, the aim of our review was to systematically summarize the anti-inflammatory activity of the plants of Clusiaceae family (sensu lato) evaluated in animal testing using predefined criteria.

## 2. Materials and Methods

### 2.1. Search Strategy for the Identification of Studies

The following databases were searched: PubMed, Scopus, and Embase, for studies reported on animals testing investigating the anti-inflammatory activity of plants of the Clusiaceae family. The electronic databases were assessed between January/2013 and May/2013. Free text searches were performed across each database to combine the terms or key words: “anti-inflammatory agents,” “Clusiaceae,” and “animals, laboratory.” The general structure of the search strategy was “anti-inflammatory agents” with the following MeSH terms or synonyms: (anti inflammatory agents) OR (agents, antiinflammatory) OR (antiinflammatories) OR (anti-inflammatory agents) OR (agents, anti-inflammatory) OR (agents, anti inflammatory) OR (anti-inflammatories) OR (anti inflammatories); “Clusiaceae” MeSH terms or synonyms: (*Psorospermum*) OR (*Psorospermums*) OR (Hypericaceae) OR (*Rheedia*) OR (*Rheedias*) OR (Plum, Waika) OR (Plums, Waika) OR (Waika Plum) OR (Waika Plums) OR (*Allanblackia*) OR (*Allanblackias*) OR (*Cratoxylum*) OR (*Cratoxylums*) (Medicinal Plant) OR (Plant, Medicinal) OR (Medicinal Plants) OR (Medicinal Herbs); and “Animals, laboratory” MeSH terms or synonyms were (laboratory animals) OR (animal, laboratory) OR (laboratory animal).

The reference list from each potentially eligible study and relevant review article was checked. The animal experiments were individually checked for inclusion criteria. Two independent researchers screened studies identified using the search strategy for inclusion; first on the basis of title and abstract and of those that were relevant, the full texts were screened for eligibility. Any disagreement was resolved through a third reviewer.

### 2.2. Inclusion and Exclusion Criteria

Preparations of Clusiaceae family administered in the animals for the treatment of inflammation were included. The following selection criteria were used for inclusion of studies in the analysis: animal studies and outcome measure. Only papers in English were included. Papers were excluded if they fulfilled one of the following criteria: (1) being not an original paper (e.g., review or letter, etc.); (2) having isolated compounds combined with plants; (3) double publication; in case a paper occurred more than one time in one of the databases, only the original manuscript was included. Purely toxicologic, analgesic, antioxidant, or other associated terms tests were not included.

### 2.3. Data Extraction Items

Items for which data were extracted include publication year, country of publication, study design, phlogistic agent, animal species, age, control groups, dose, duration, number of animal evaluated in each group, anti-inflammatory effect, outcome measurement tools, and author's conclusions.

## 3. Results and Discussion

**Figure 1:**
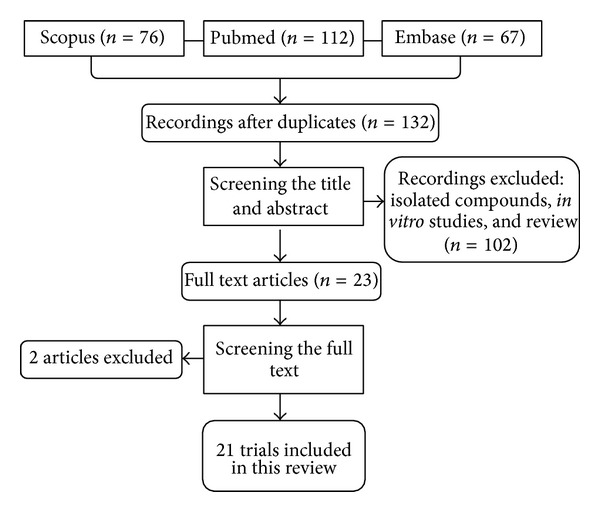
Flow diagram of the literature search.

As illustrated in the flow diagram, of the all unique records identified, only 23 publications met criteria for full-text review. We screened 255 relevant articles, and 233 were excluded, leaving us with 23 full-text eligible articles. Of these, 2 more were excluded (Figure 1). Characteristics of included articles are summarized in Table 1.

From the search, 29 hits were found with different Clusiaceae species reporting one or more of these activities: antinociceptive, anti-inflammatory, and antipyretic activity and gastric and toxicology effects. Some of the reports coincide for a given species, and, therefore, a total of 19 plants were reported to have such activity. However, 11 plants were studied for such activity. In eleven cases, further phytochemical studies were carried out to find out the active constituent(s). The Clusiaceae plant names mentioned in this review were taken textually from the original sources, whenever they were reported.

The species were able to significantly reduce the inflammatory response in several models with possible involvement of isolated compounds: genus* Allanblackia*:* A. gabonensis* Sosef & Dauby and* A. monticola* Staner L. C.; genus* Clusia*:* C. nemorosa G. *Mey.; genus* Garcinia* or* Rheedia*:* G. brasiliensis* Mart.,* G. cambogia *Desr.,* G. gardneriana *(Planchon & Triana) Zappi.,* G. hanburyi* Hook F., and* Rheedia longifolia* Planch & Triana; genus* Hypericum*:* H. androsaemum* L.,* H. barbatum *Jacq.,* H. canariense* L.,* H. empetrifolium* Willd.,* H. glandulosum *Dryand. Ait,* H. hirsutum* L.,* H. perforatum* L.,* H. reflexum *L. f.,* H. richeri* Vill.,* H. rumeliacum* Boiss. subsp.* apollinis *(Boiss. & Heldr.) Robson & Strid, and* H. triquetrifolium* Turra.

In all studies the minimum information reporting research using animals was included, such as the number and specific characteristics of animals used (including species, strain, sex, and genetic background); details of housing and husbandry; and the experimental and statistical methods.

Since animal models are fundamental tools in biomedical study, as the ones of sharing a high degree homology with humans, mice and rats are commonly used in laboratory tests for better understanding human disorders. As mammals, murine models with drug-induced diseases have been well-established, either for investigating disease pathogenesis and probable mechanisms, or for assessing the effectiveness of diverse candidate instruments and drugs, physically and chemically, which facilitated human health researches [30, 31].

The animals most used were* Swiss* mice,* Wistar*, and* Sprague-Dawley* rats of both genders. Animals studied were approximately 3 to 6 weeks of age. Altogether, 12 animal experiments or experimental settings matched inclusion criteria. Often, experiments consisted of several subexperiments with safety results usually reported globally; accordingly, these subexperiments are summarized in this review as well. The inflammation tests differed, but in general the tests were based on sensitization with doses of injections of inflammatory agents at different frequencies and for an average duration of hours or days.

### 3.1. Plants of Genus* Allanblackia*

Anti-inflammatory effects of* A. gabonensis* stem bark aqueous extract on carrageenan, histamine, and serotonin-induced paw edema were assessed. The aqueous extract on serotonin, histamine, or carrageenan-induced edema showed a significant inhibition starting from the first hour up to the sixth hour. On paw edema induced by histamine and serotonin, the significant reduction with a maximal inhibition of 56.94% and 40.83% was observed, respectively [19].

Other species evaluated were* A. monticola* on carrageenan-induced edema and demonstrated that the methylene chloride extract and methylene chloride/methanol extract and its methanol fraction showed maximum reductions and a maximum inhibition of paw edema. The methylene chloride fraction of* A. monticola* on rat paw edema induced by histamine exhibited a significant reduction of inflammation. The fraction did not reveal an anti-inflammatory activity even at the highest concentration in serotonin test. This same fraction on paw edema induced by arachidonic acid inhibited the paw edema. Though being on dextran-induced paw edema the fraction decreased the volume an hour later compared with control groups [16].

*Allanblackia gabonensis* and* Allanblackia monticola *exhibited significant activity against edematous effect in all the three phases [16, 19], involved in the release of serotonin and histamine and mediated by prostaglandins, cyclooxygenase products, and the phase provided by kinins. Moreover, there is evidence of a possible interaction of* A. gabonensis* extract with the liberation and/or action of endogenous histamine and serotonin, probably mediated by alkaloids, phytosterols, triterpenes, and phenols compounds isolated, such as xanthones and triterpenes on the genus* Allanblackia* [32, 33].

For the same animal protocol utilizing histamine and serotonin,* A. monticola* was able to inhibit edema induced by dextran and histamine but not that provoked by serotonin. Probably the result on edema provoked by arachidonic acid indicated that this occurs preferentially by inhibition of lipoxygenase pathway of arachidonate metabolism [16]. Previous studies demonstrated that cytotoxic, anti-inflammatory, antimicrobial, antifungal, and HIV inhibitory activities of species of this genus are due to many of the secondary metabolites, xanthone derivatives (allanxanthone B, allanxanthone C, rubraxanthone, tovophyllin A, garciniafuran, norcowanin, and mangostin), pentacyclic triterpene (lupeol), saponin (a 3-O-*β*-D-glucopyranoside of stigmasterol), and phytosterol (stigmasterol) [34, 35].

### 3.2. Plants of Genus* Clusia*

Hexane extract of leaves from* C. nemorosa* was evaluated on carrageenan-induced pleurisy. The extract caused a significant decrease in total protein extravasations, decreased the volume of the exudates and inhibited leukocyte migration. A significant reduction in TNF-*α* concentration was verified in the treated group with hexane extract. The granuloma formation response elicited by subcutaneously implanted cotton pellet was inhibited [36].

Farias et al. (2011) report these actions to carry out experimental protocols on animals such as the carrageenan-induced pleurisy, a model widely used to investigate the pathophysiology of acute inflammation and also for evaluating the efficacy of drugs in inflammation [36]. The presence of carrageenan in the pleural cavity attenuates the plasma extravasation by increasing also the amount of total leukocytes especially neutrophils and mononuclear cells. After the fourth time, there is also a significant increase in the levels of TNF-*α* on the site that received the injury with a marked release of histamine and serotonin [37–39]. In the parameters involved in the pathological process, the* C. nemorosa* Both acted in significant reduction of leukocyte migration, with emphasis on the reducing levels of neutrophils, with proven results* in vitro* protocols using as the initiator CXCL1 [36].

These results are in agreement with the findings of Ferro et al. (2013), suggesting that the mechanism of* C. nemorosa* may be linked, in part, to the inhibition of cyclooxygenase and/or lipoxygenase products in inflammatory diseases mediated by peripheral mechanisms [40]. The ratification of the anti-inflammatory effects of this plant occurred with the results obtained in the formalin test, a model of inflammatory pain that has two distinctive phases. The first phase corresponding to neurogenic pain is caused by activation of sensory C-fibers, followed by a second stage which is associated with the development of an inflammatory mediator release [41]. It is established that histamine, serotonin, prostaglandins, and bradykinin are involved in the second phase responses [42]. With the findings of this experiment, pelleted better anti-inflammatory properties of* C. nemorosa*, therefore only in the inflammatory phase species under study showed significant results.

Chemical studies carried out with some species belonging to the genus* Clusia* have demonstrated the presence of many constituents, including polyisoprenylated benzophenones, terpenes, benzoquinone, flavonoids, dihydrophenanthrene derivative, tocotrienolic acids, betulinic acid, kaempferol, and sitosterol glucoside [43]. The anti-inflammatory activity of this genus can be attributed to these compounds. For betulinic acid, an approach of the action mechanism is attributed to its effect on NF-*κ*B through inhibition of I*κ*B kinase and p65 phosphorylation [44].

### 3.3. Plants of Genus* Garcinia*

The anti-inflammatory effect of the leaves extract from* G. brasiliensis* on carrageenan-induced rat paw edema or peritonitis induced by lipopolysaccharide or granulomatous tissue growth induced cotton pellet implantation was tested. The leukocyte recruitment at 4 h after LPS was 27.9%, 51.5%, and 55.8% for 30, 100, and 300 mg/kg of the extract, respectively. In the model of chronic inflammation using cotton pellet-induced fibrovascular tissue growth in rats, the extract significantly inhibited the formation of granulation tissues [23, 45].

One study reported that the hydroalcoholic extract of* G. gardneriana* was evaluated on carrageenan, 12-O-tetradecanoylphorbol-acetate (TPA), or different inflammatory mediators, including bradykinin, substance P, histamine, prostaglandin E_2_, or arachidonic acid measurement of paw edema. The activity of tissue myeloperoxidase (MPO) was assessed after injection of carrageenan into the mouse right hindpaw. All of the tested extracts from leaves, bark, and seeds presented an inhibitory effect on the edema induced by carrageenan. The extract from leaves produced a significant reduction in the mouse paw edema induced by most tested mediators, except for the AA-induced edema. On MPO activity, treatment with extracts from leaves, bark, and seeds of* G. gardneriana* significantly prevented the increase in MPO activity induced by carrageenan [21, 28].

Ethyl phenylpropiolate- (EPP-) induced ear edema was utilized for testing inflammatory activity topical of the ethyl acetate extract from* G. hanburyi*, which at the dose of 1 mg per ear significantly inhibited the edema formation. The paw edema was produced in rats by either carrageenan or arachidonic acid (AA). The extract markedly reduced the edema formation of the paw induced by carrageenan at all assessment times; however, it is not elicited inhibitory effect on the edema formation of the rat paw induced by AA. For test cotton pellet-induced granuloma formation the ethyl acetate extract from* G. hanburyi* significantly reduced transudative weight and granuloma formation [20].

The administration of* G. cambogia* extract reduced the length of macroscopically observed lesions at a 1 g/kg dosage in colitis, although the MPO activity was significantly reduced by* Garcinia* treatment. The* G. cambogia extract* effectively reduced colonic IL-1*β* expression and was also effective in inhibiting the iNOS colonic expression induced by 2,4,6-trinitrobenzene sulfonic acid (TNBS). It was found that the administration of the extract caused a substantial reduction in the COX-2 expression, as well as in the upregulation of PGE_2_ caused by TNBS in the colon [24].

The fruit-peel volatile oil of* G. brasiliensis* was evaluated on the induced gradual edema of rat paw upon application of the inflammatory agent carrageenan. The inflammatory process was inhibited after administration of carrageenan [23].

The leaves aqueous extract of* Rheedia longifolia* inhibited inflammation six hours after the intrathoracic administration of LPS in the pleural wash recovered from LPS-injected mice [29].

These studies for* Garcinia* (*Rheedia*) species revealed the* G. gardneriana *like being effective in reducing the edematogenic response. This effect is maybe related to a reduction in the liberation of histamine, serotonin, or bradykinin in local tissue or due to the blockage of receptors to these different mediators. This species significantly reduced leukocyte migration and decreased the dry weights of implanted cotton pellets, suggesting the potential to reduce the number of fibroblasts and the synthesis of collagen and mucopolysaccharides, probably by action of the active anti-inflammatory agents [45]. It is suggested that many species of this genus possess anti-inflammatory and analgesic activity in many animal models [20].

Recently, it was showed that 7-epiclusianone, a polyisoprenylated benzophenone naturally found in the fruit of* G. brasiliensis* or isolated from* G. gardneriana* [46], presents several biological effects, such as antibacterial* in vivo* [47–49]. Volatile oils exhibit of* G. brasiliensis *presented biological activities such as antiviral, antibacterial, and anti-inflammatory properties [23]. The pharmacological study of polyisoprenylated benzophenones has been shown to be of interest due to the wide spectra of activities attributed to its derivatives [9, 50]. Considering the studies already described and the polyisoprenylated benzophenones in* Garcinia* species, studies confirmed the probable anti-inflammatory activity of 7-epiclusianone [51].

The species* Rheedia longifolia* inhibits neutrophil accumulation in the pleural cavity of mice, which is indicative of its anti-inflammatory activity. In addition, the aqueous crude extract also shows antinociceptive activity similar to that of an opioid agonist [29]. The* Rheedia* genus is characterized by the presence of triterpenes, steroids, coumaric acid, xanthones, and benzophenones and it is interesting that only the butanol and aqueous fractions inhibited inflammatory nociception, a characteristic of Arylpropanoids that is not observed in the dichloromethane and ethyl acetate fractions. The Arylpropanoids group may be responsible for the inhibition of neurogenic nociception [52].

### 3.4. Plants of Genus* Hypericum*

Topical anti-inflammatory activity of the infusion, methanol extract, and fractions of the aerial part in blossom of* H. canariense* L. and* H. glandulosum* Ait. in mice were verified in one study. It was observed that all extracts assayed, with the exception of the infusions of both species and the* H. canariense* aqueous fraction, showed a significant inhibition of the TPA-induced ear edema in a dose dependent manner as compared to control. The* H. canariense* methanol extract and* H. glandulosum* butanol fraction at 1 mg/ear were the most effective [13].

The anti-inflammatory activity of the total ethanol extracts of* H. perforatum* and some other* Hypericum* species was by using the carrageenan-induced rat paw edema test. The results indicated that all examined extracts (*H. androsaemum*,* H. hirsutum*,* H. richeri*,* H. perforatum*, and* H. barbatum*) possessed anti-inflammatory activity, especially the dry extracts of* H. hirsutum* and* H. perforatum* [17]. For the species* H. empetrifolium*, the methanolic extract administered showed a significant antiedemic effect on carrageenan-induced paw edema in rats from the first hour until the third hour, when the inhibitory effect was greatest [11].

Other member of this genus,* H. rumeliacum* Boiss. subsp. apollinis (Boiss. & Heldr.) Robson & Strid, presented anti-inflammatory activity of the methanol extract in the experimental model of only at a dose of 70 mg/kg. The effect was significant from the first to the third hour [22].

The effect of an acute administration of* H. perforatum* was verified on carrageenan-induced paw edema. As a result,* H. perforatum* dose dependently inhibited the carrageenan-induced inflammatory edema with maximal effect 1 h after carrageenan injection [14]. Another study for the same species demonstrated effect for the treatment of active inflammatory periodontal disease, also it was demonstrated that* Hypericum* exerts a significant inhibitory effect on plasma extravasation and reduced the degree of bone resorption during periodontitis [26]. Zdunić et al. (2009) investigated the anti-inflammatory activity in rats by administration of* H. perforatum* oil extracts on carrageenan-induced rat paw edema with significantly inhibition by all three tested oil extracts [25]. More recently, Süntar et al. (2010) evaluated acetic acid-induced increase in capillary permeability of the samples of* H. perforatum* in mice. A dose-dependent inhibitory activity was observed for ethanolic extract up to the dose of 200 mg/kg with the highest inhibitory value of 40.9% and also was exerted by extract and its fractions significant and dose-dependent anti-inflammatory activity [27].

A single study showed the anti-inflammatory activity of the total extract of* H. triquetrifolium*, evaluated by the carrageenan-induced paw edema test in the rat and was able to inhibit paw swelling dose-dependently after carrageenan injection [12].

Topical anti-inflammatory activity obtained from TPA-induced mice ear edema test was demonstrated for the infusion, methanol extract, and different fractions from* H. reflexum*. It was observed that all extracts assayed, with the exception of the infusion and the aqueous fraction, showed a significant inhibition of ear edema in a dose-dependent manner [15].

These results show that ten studies revealed a decrease in inflammation of species of genus* Hypericum*. Oil extract of* H. perforatum* showed the highest activity probably due to the greatest amount of quercetin and I3,II8-biapigenin, and both compounds administered showed anti-inflammatory activity [25]. Moreover, studies demonstrated that quercetin produced an anti-inflammatory effect on the acute inflammation [53, 54]. More recently, it was suggested the anti-inflammatory effect of* H. perforatum* could interfere with the actions of histamine, serotonin, or kinins and to reduce cells infiltration, mediated by downregulating adhesion molecules ICAM-1 and P-selectin [55]. Menegazzi et al. (2006) showed that the anti-inflammatory activity of* H. perforatum* might be due to the inhibition of nuclear factor-kappa B and STAT-3 activation [56]. Study* in vitro* evidenced that the flavonoids, such as quercetin, along with pseudohypericin and hyperforin might be the major anti-inflammatory components of this species [57] and able to inhibit the production of proinflammatory mediators such as prostaglandin E_2_ (PGE_2_), tumor necrosis factor-*α* (TNF-*α*), and interleukin-10 (IL-10).

These researches are according to an investigation of the effect of* H. perforatum* on the NF-*κ*B inflammation factor, conducted by Bork et al. (1999), in which hyperforin provided a potent inhibition of TNF*α*-induced activation of NF-*κ*B [58]. Another important activity for hyperforin is a dual inhibitor of cyclooxygenase-1 and 5-lipoxygenase [59]. Moreover, this species attenuated the expression of iNOS in periodontal tissue, which may contribute to the attenuation of the formation of nitrotyrosine, an indication of nitrosative stress [26]. In this context, a combination of several active constituents of* Hypericum* species is the carrier of their anti-inflammatory activity.

The topical treatmentof* H. canariense* and* H. glandulosum* inhibited TPA-induced ear edema in mice, indicating the presence of active substances endowed with anti-inflammatory activity. The active principle responsible for the anti-inflammatory-like effects of these species is/are, so far, not known, but preliminary phytochemical analysis carried out with the methanol extract of both species revealed the presence of flavonoids, tannins, and anthraquinones [60]. In the same topical model of inflammation,* H. reflexum* inhibited the edema probably by the presence of tannins, flavonoids, saponins, and anthraquinones in this species, as reported in previous studies [15, 53, 61, 62].

A single study showed the anti-inflammatory effects of* H. barbatum*,* H. androsaemum*,* H. richerii*,* H. hirsutum*, and* H. perforatum *produced significant dose-dependent anti-inflammatory effect which was not correlated with the hypericin content in these extracts. It suggests the involvement of other active substances, besides hypericin, in the anti-inflammatory effect of* Hypericum* species tested [17].* H. rumeliacum* subsp. apollinis methanol extract administration inhibited the paw edema and reduced the infiltrates, both between connective fibres and into intercellular spaces [22].

The* H. empetrifolium* was also reviewed in this study due to significant results in experimental model of inflammation. It has been mentioned above that acute inflammation caused by carrageenan is characterized by a biphasic event and various mediators such as histamine, serotonin, bradykinin, and substance P release, and later by infiltration of PMN cells at the site of inflammation which induces secretion of various pro-inflammatory mediators such as nitric oxide, prostaglandins, and cytokines [38, 63]. Within this context, the anti-inflammatory action of* H. empetrifolium* may be related to the inhibition of prostaglandin synthesis.

Some of the studies had insufficiently described methods for detecting this effect or scantily reported the results. The findings include a decrease of total leukocytes, a number of neutrophils, total protein concentration, granuloma formation, and paw or ear edema formation. Other interesting findings included the decrease of the MPO activity, inflammatory mediators such as NF-*κ*B and iNOS expression and PGE_2_ and Il-1*β* levels and a decrease in chronic inflammation. This variability can result in significant differences in anti-inflammatory activity, making it difficult to the limitations in your study. Due to the limited number of animal experiments included in some studies, we cannot draw definitive conclusions; however, the results allow us to believe in the potential of these plants as anti-inflammatory agents.

## 4. Conclusion

Taking all results collectively, plants of Clusiaceae family were found to have acceptable anti-inflammatory profiles. The isolation and purification of the chemical constituents from these plants and subsequent evaluation of their pharmacologic effects contribute to its anti-inflammatory effect understanding. Therefore, this family should attract the interest of researchers for clinical and toxicological studies, as well as for the herbal pharmaceutical industry. More studies with other methodological in order to investigate the quality of these plants are needed.

## Figures and Tables

**Table 1:** Description of the anti-inflammatory aspects of the studies included in systematic review.

References	Scientific name	Parts used	Animal	Dose	Route	Methods used	Action mechanism
Trovato et al., 2001 [11]	*H. empetrifolium *	APME	Wistar rats	100 mg/kg	i.p.	CIPE	Inhibition of PG
Ozturk et al., 2002 [12]	*H. triquetrifolium *	DAPE	Wistar rats	25–60 mg/kg	i.p.	CIPE	Without mechanism
Rabanal et al., 2005 [13]	*H. canariense; H. glandulosum *	BME	Swiss mice	0.25–1 mg/ear	t.a.	TPAIEE	Inhibition of AA metabolism
Abdel-Salam 2005 [14]	*H. perforatum *	Commercial extract	Sprague-Dawley rats	50–300 mg/kg	s.c.	CIPE	Inhibition of the liberation of HIS, 5-HT, KN
Sánchez-Mateo et al., 2006 [15]	*H. reflexum *	BME	Swiss mice	0.25–1 mg/ear	t.a.	TPAIEE	Inhibition of PLA_2_, COX, and LOX
Nguemfo et al., 2007 [16]	*A. monticola *	SBMCME	Wistar rats	75–300 mg/kg	p.o.	CIPE; HSIPE; AAIPE; DIPE	Inhibition of AA metabolism
Šavikin et al., 2007 [17]	*H. perforatum; H. barbatum; H. hirsutum; H. richeri; H. androsaemum. *	DAPE	Wistar rats	25–200 mg/kg	p.o.	CIPE	Inhibition of NF-*κ*B.
Frutuoso et al., 2007 [18]	*R. longifolia *	LAE	Swiss mice; Wistar rats	10–100 mg/kg	p.o.	PILPS	Inhibition of neutrophil
Ymele et al., 2013 [19]	*A. gabonensis *	SBAE	Wistar rats	100–400 mg/kg	p.o.	CIPE; HSIPE	Reduced liberation and action of His and 5-HT; inhibition AA metabolism
Šavikin et al., 2007 [17]	*H. perforatum; H. barbatum; H. hirsutum; H. richeri; H. androsaemum. *	DAPE	Wistar rats	25–200 mg/kg	p.o.	CIPE	Inhibition of NF-*κ*B.
Panthong et al., 2007 [20]	*G. hanburyi *	BEAE	Sprague-Dawley rats	10–40 mg/kg	p.o.	EPPIEE; CIPE; AAIPE; GGICP	Inhibition of the liberation of His, PG, KN
Castardo et al., 2008 [21]	*G. gardneriana *	LHE	Swiss mice	30–300 mg/kg	i.p.	CIPE; HSIPE; TPAIPE; BKIPE; AAIPE MPOAA; SPIPE	Inhibition of the activity of neuropeptides and PKC
Galati et al., 2008 [22]	*H. rumeliacum *	APME	Wistar rats	50; 70 mg/kg	i.p.	CIPE	Without mechanism
Martins et al., 2008 [23]	*G. brasiliensis *	FPO	Wistar rats	100 mg/kg	p.o.	CIPE	Without mechanism
dos Reis et al., 2009 [24]	*G. cambogia *	FPE	Wistar rats	0.5; 1.0 g/kg	p.o.	CITNBS; MPOAA; EMPGE_2_	Inhibition COX-2 expression and production of PGE_2_
Zdunić et al., 2009 [25]	*H. perforatum *	FTOE	Wistar rats	1.25 mL/Kg	p.o.	CIPE	Without mechanism
Paterniti et al., 2010 [26]	*H. Perforatum *	ME	Sprague-Dawley rats	2 mg/kg		PIL; MPOAA; MVP; CE	Reduces the NF-*κ*B translocation; inhibition the I*κ*B-*α* degradation; attenuation of the expression of iNOS
S$\ddot{\text{u}}$ntar et al., 2010 [27]	*H. perforatum *	APOOE; APEE	Sprague-Dawley rats; Swiss mice	50–400 mg/kg	p.o.	AcAICP	Without mechanism
Otuki et al., 2011 [28]	*G. gardneriana *	LHE; BHE; SHE	Swiss Webster mice	0.01–1 mg/ear	t.a.	COIEE; MPOAA	Inflammatory signal transduction pathway not specified
Ozturk et al., 2011 [12]	*G. brasiliensis *	LEE	Wistar rats	30–300 mg/kg	p.o.	CIPE; PILPS; GGICP	Inhibition of the liberation of His, 5-HT, and BK
Santos et al., 2011 [29]	*C. nemorosa *	LHxE	Swiss mice	50–200 mg/kg	i.p.	CIP; MTP; MTNF-*α*; GGICP	Inhibition of the neutrophil migration

*Abbreviations of parts used*: APME: aerial parts methanol extract; APEE: aerial parts ethanolic extract; APOOE: aerial parts olive oil extract; BEAE: bark ethyl acetate extract; BHE: bark hydroalcoholic extract; BME: blossom methanol extract; DAPE: dried aerial parts extract; FPE: fruit peel extract; FPO: fruit pee oil; FTOE: flowering tops oil extracts; LAE: leaves aqueous extract; LHE: leaves hydroalcoholic extract; LEE: leaves ethanolic extract; LHxE: leaves hexanic extract; ME: methanolic extract; SBAE: stem bark aqueous extract; SBMCME: stem barks methylene chloride/methanol extract; SHE: seeds hydroalcoholic extract.

*Abbreviations of administration routes*: i.p.: intraperitoneal; p.o.: oral administration; t.a.: topical administration.

*Abbreviations of methods used*: AAIPE: arachidonic acid-induced paw edema; AcAICP: acetic acid-induced increase in capillary permeability; BKIPE: bradykinin induced paw edema; CE: cytokines expression; CIP: carrageenan-induced pleurisy; CIPE: carrageenan-induced paw edema; CITNBS: colitis induced by 2,4,6-trinitrobenzenesulfonic acid; DIPE; dextran-induced paw edema; COIEE: croton oil-induced ear edema; EMPGE_2_: evaluation of mucosal PGE_2_; EPPIEE: ethyl phenylpropiolate induced ear edema; GGICP: granulomatous growth induced by cotton pellet; HSIPE: histamine and serotonin induced paw edema; MPOAA: myeloperoxidase activity assay; MTNF-*α*: measurement of tumor necrosis factor alpha; MTP: measurement of total protein; PEILPS: peritonitis induced by lipopolysaccharide; PIL: peritonitis induced by ligature; PILPS: pleurisy induced by LPS; SPIPE: substance P induced paw edema; TPAIPE: 12-O-tetradecanoylphorbol 13-acetate induced paw edema.

*Abbreviations of action mechanism*: AA: arachidonic acid; BK: bradykinin; COX: cyclooxygenase; His: histamine; iNOS: inducible nitric oxide synthase; KN: kinins; LOX: lipoxygenase; MVP: measurement of vascular permeability; NF-*κ*B: nuclear factor kappa B; PKC: protein kinase C; PLA2: phospholipase A2; PG: prostaglandin, 5-HT: 5-hydroxytryptamine.
